# HINN prioritizes multi-omics biomarkers associated with cognitive decline

**DOI:** 10.1093/bioinformatics/btag741

**Published:** 2026-10-07

**Authors:** Yashu Vashishath, Sarah Anne Beaver, Fahad Saeed, Serdar Bozdag

**Affiliations:** Department of Computer Science and Engineering, University of North Texas, Denton, TX 76203, United States; Center for Computational Life Sciences, University of North Texas, Denton, TX 76203, United States; BioDiscovery Institute, University of North Texas, Denton, TX 76203, United States; Department of Computer Science and Engineering, University of North Texas, Denton, TX 76203, United States; School of Computing and Information Sciences, Florida International University, Miami, FL 33199, United States; Department of Computer Science and Engineering, University of North Texas, Denton, TX 76203, United States; Center for Computational Life Sciences, University of North Texas, Denton, TX 76203, United States; BioDiscovery Institute, University of North Texas, Denton, TX 76203, United States; Department of Mathematics, University of North Texas, Denton, TX 76203, United States

## Abstract

**Motivation:**

Integrating multi-omics data for disease prediction remains challenging due to the complexity of cross-layer biological interactions and the limited use of prior biological knowledge in many machine learning models. We present the Hierarchical Input Neural Network (HINN), a deep learning framework that incorporates biologically informed connections across genomic, epigenetic, and transcriptomic layers to model regulatory relationships in a structured and interpretable manner. HINN was applied to blood-derived multi-omics data to predict cognitive assessment scores, including Mini-Mental State Examination (MMSE), Montreal Cognitive Assessment, Alzheimer’s Disease Assessment Scale–Cognitive Subscale 11 tasks (ADAS11), and Rey Auditory Verbal Learning Test. A biologically guided feature construction pipeline was used to integrate genome-wide association studies-derived single nucleotide polymorphisms (SNPs), promoter-region DNA methylation, and gene expression data through Gene Ontology Biological Process and KEGG pathway mappings.

**Results:**

The model was evaluated using a rigorous training strategy that separates feature selection from testing and incorporates cross-validation as well as repeated runs to ensure robustness. Across all cognitive outcomes, HINN achieved the lowest mean normalized mean squared error among the evaluated models, with statistically significant improvements over most baseline methods. Ablation analyses showed that the contributions of individual omics modalities and biologically informed connectivity varied across cognitive outcomes, with the clearest benefits of structured connectivity observed for ADAS11 and MMSE. Feature attribution analysis identified a multi-omics cascade involving SNP (rs116557230), CpG site (cg24041822), and a *SOCS4* gene expression probe, which showed consistent associations with multiple cognitive outcomes. These findings highlight the ability of HINN to uncover biologically meaningful cross-omics relationships relevant to cognitive decline. Together, this work demonstrates that integrating deep learning with biological knowledge enables biomarker discovery for complex diseases.

**Availability and implementation:**

All code, sample data, and instructions are publicly available via GitHub (https://github.com/bozdaglab/HINN) and archived on Zenodo (https://doi.org/10.5281/zenodo.19197057) to ensure reproducibility and long-term accessibility.

## 1 Introduction

Understanding and predicting complex diseases remains a fundamental challenge in healthcare. In recent years, multi-omics data combined with machine learning (ML) approaches have been used increasingly to characterize biological systems and improve disease prediction (Hasin *et al.* 2017, Ren *et al.* 2025). Individual omics layers provide complementary information, and their integration enables a more comprehensive view of molecular mechanisms underlying disease. ML models are well suited for this task, as they can capture complex patterns in high-dimensional data that are difficult to model using traditional statistical approaches (Zhavoronkov *et al.* 2019).

The integration of multi-omics data has shown promise for disease prediction and biomarker discovery, including early detection, identification of molecular signatures, and support for personalized treatment strategies (Yang *et al.* 2024, Ozdemir *et al.* 2025). By jointly modeling multiple molecular layers, these approaches can improve prediction of disease susceptibility, progression, and therapeutic response.

Deep neural networks (DNNs) have emerged as a powerful tool for modeling complex biological data (Webb 2018, Zhavoronkov *et al.* 2019). More recently, efforts have focused on incorporating prior biological knowledge into neural network (NN) architectures to improve interpretability and biological relevance (Picard *et al.* 2021). For example, Deng *et al.* (2020) introduced the pathway-guided neural network (PGNN), which integrates pathway information into a DNN framework. Similarly, P-NET organizes gene-level molecular profiles through hierarchical biological pathways to enhance interpretability (Elmarakeby *et al.* 2021). Graph-based approaches, such as Gene-level biomarker discovery from multi-Omics data using graph ATtention neural network (GOAT), have also been proposed to capture gene–gene interactions using learned representations (Jeong *et al.* 2023, Ozdemir *et al.* 2025).

Despite these advances, existing approaches have limitations in modeling cross-omics regulatory relationships. Many methods focus on interactions within a single molecular layer or aggregate multiple omics modalities without explicitly capturing structured dependencies between them. However, biological regulation is inherently hierarchical across omics levels. For example, DNA methylation can influence gene expression (Razin and Cedar 1991, Jones *et al.* 2013), and single nucleotide polymorphisms (SNPs) can affect both methylation patterns and transcriptional activity (Vohra *et al.* 2020). These cross-layer relationships vary across individuals and are often not explicitly modeled in existing frameworks (Cao and Gao 2022).

To address these limitations, we propose Hierarchical Input Neural Network (HINN), a deep learning framework that incorporates biologically grounded cross-omics relationships directly into its architecture. HINN models regulatory dependencies between SNPs, nearby CpG methylation sites, and gene expression using biologically constrained connections, enabling structured integration of multi-omics data (Fig. 1). We further compare HINN against pathway-guided and graph-based architectures, including PGNN and GOAT, to evaluate the impact of hierarchical modeling.

**Figure 1:**
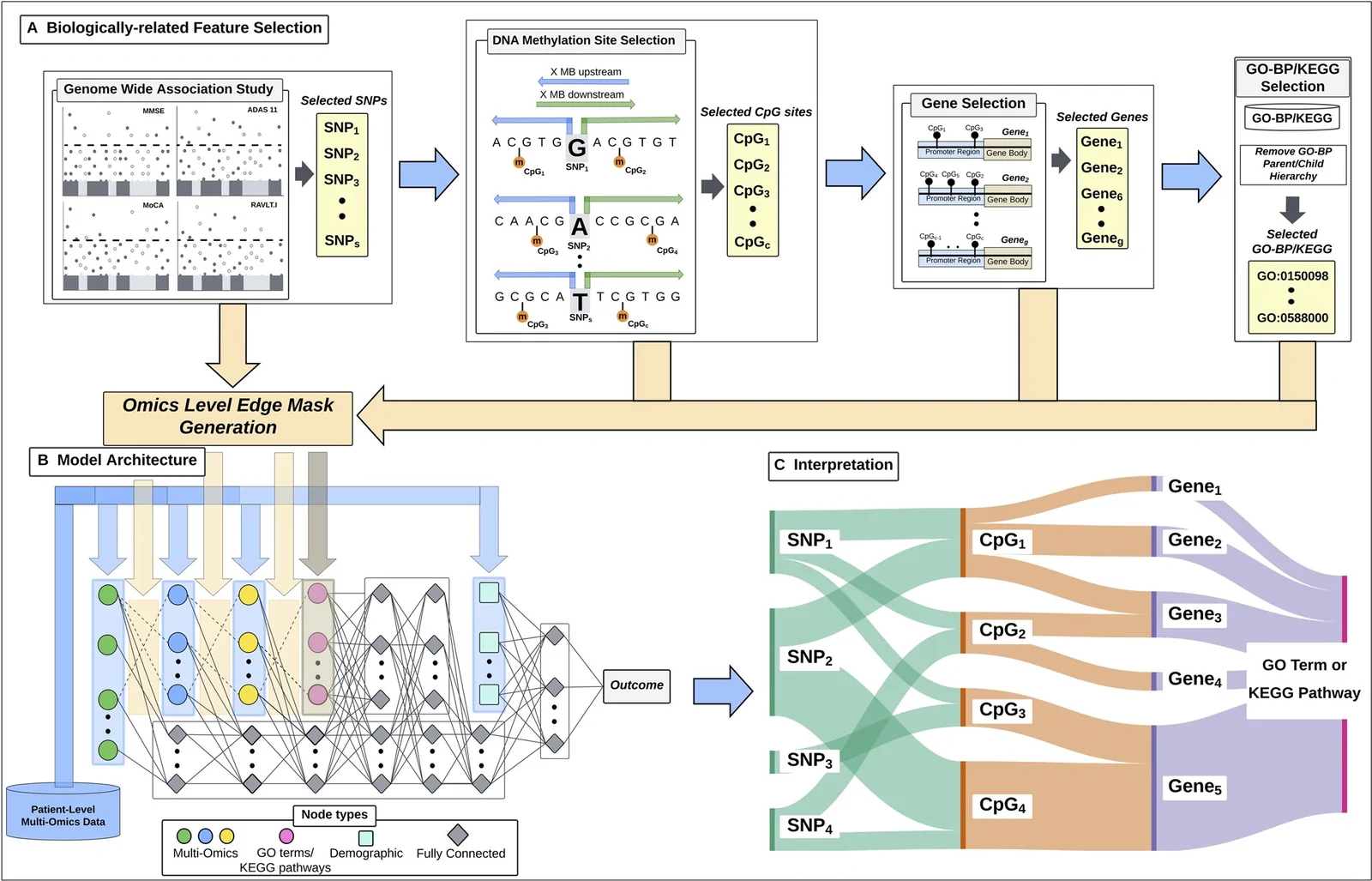
Overview of the HINN framework. (a) The biologically informed feature selection process begins by identifying significant SNPs via GWAS. CpG sites located within a ±1 Mb window around the significant SNPs and overlapping promoter regions are selected. Genes linked to these CpG sites are then retained, and their associated GO-BP terms and KEGG pathways are incorporated without enrichment-based filtering. (b) HINN integrates multi-omics data (SNPs, DNA methylation, and gene expression) using a hierarchical architecture. Each data modality is represented as a distinct input layer, with biologically meaningful interconnections across layers. Demographic information is incorporated into the model at later stages. (c) The model’s interpretability is enhanced by visualizing key predictive features across omics layers.

As a case study, we applied HINN to blood-derived multi-omics data to predict cognitive function using standardized assessments, including the Mini-Mental State Examination (MMSE) (Folstein *et al.* 1975), Montreal Cognitive Assessment (MoCA) (Nasreddine *et al.* 2005), Alzheimer’s Disease Assessment Scale–Cognitive Subscale 11 tasks (ADAS11) (Samtani *et al.* 2015), and Rey Auditory Verbal Learning Test (RAVLT.Immediate) (Bean 2011). These assessments are widely used to evaluate cognitive function and support the clinical assessment of neurodegenerative conditions such as Alzheimer’s disease (AD) and mild cognitive impairment (MCI), yet their clinical interpretation can be subjective and limited in sensitivity (Daffner *et al.* 2015, Kelso and Tadi 2022).

Our results show that HINN improves predictive performance while enabling biologically interpretable insights. In particular, the model identifies multi-omics feature cascades linking genetic variation, epigenetic regulation, and gene expression, with individual components showing significant associations with cognitive outcomes. Overall, this framework bridges deep learning and biological knowledge to support interpretable, blood-based biomarker discovery for cognitive function. All code, sample data, and instructions are publicly available via GitHub (https://github.com/bozdaglab/HINN) and archived on Zenodo (https://doi.org/10.5281/zenodo.19197057) to ensure reproducibility and long-term accessibility.

## 2 Methods

### 2.1 Dataset

Data used in the preparation of this article were obtained from the Alzheimer’s Disease Neuroimaging Initiative (ADNI) database (http://adni.loni.usc.edu). The ADNI was launched in 2003 as a public–private partnership, led by Principal Investigator Michael W. Weiner, MD. The primary goal of ADNI has been to test whether serial magnetic resonance imaging, positron emission tomography, other biological markers, and clinical and neuropsychological assessment can be combined to measure the progression of MCI and early AD (Jack Jr *et al.* 2008, Weiner *et al.* 2015).

The study utilized data modalities, including cognitive assessments (MMSE, MoCA, ADAS11, and RAVLT.Immediate), genome-wide association studies (GWAS)-derived SNPs, DNA methylation, and gene expression data. Demographic variables (age, gender, race, and education), APOE4 genotype, and plasma p-Tau 181 levels were also incorporated. After removing samples with missing data across modalities, 452 individuals remained. The dataset was split into training (*n* = 316) and held-out test (*n* = 136) sets. All feature selection, genomic window-size selection, hyperparameter optimization, and model training procedures were performed using only the training data. The held-out test set was excluded from these procedures and was used only for final model evaluation (Supplementary Information, available as supplementary data at *Bioinformatics* online).

### 2.2 Mathematical foundation of the HINN architecture

To formalize the biological relationships in our hierarchical network, we developed a mathematical framework that governs feature interactions across different omics layers. The core of our approach lies in creating structured connections between layers that reflect biologically informed dependencies across molecular levels. Figure S1A, available as supplementary data at *Bioinformatics* online, illustrates the overall architecture and information flow.

#### 2.2.1 Mask-based connection framework

Biologically supported connections between omics layers are represented using a mask-based connection framework. This mechanism uses a binary mask matrix $M_{l}$ for each layer *l* to specify permitted interactions between the input and output features of a given layer.

Let the input to layer *l* be denoted as $X_{l}\in R^{c\times d}$, where *c* is the number of samples and *d* is the number of input features. The corresponding output is represented as $Y_{l}\in R^{c\times e}$, where *e* denotes the output feature dimension. The transformation is parameterized by a weight matrix $W_{l}\in R^{d\times e}$ and a bias vector $P_{l}\in R^{e}$.

The transformation from one layer to the next is defined by the masked linear mapping:


(1) $$f(X_{l},M_{l})=\sigma \left(X_{l}\cdot (M_{l}⊙W_{l})+P_{l}\right)\;$$

Equation (1) defines the core masked transformation used throughout the network. The binary mask $M_{l}\in {\left\{0,1\right\}}^{d\times e}$ in layer *l* restricts connections to biologically plausible interactions, encoding biologically informed relationships, including SNP–CpG proximity, promoter mapping, and gene–pathway annotations derived from Gene Ontology Biological Process (GO-BP) and KEGG.

In Equation (1), ⊙ denotes element-wise (Hadamard) multiplication, which ensures that only connections allowed by the mask $M_{l}$ are retained. The matrix product computes the masked transformation of the input, followed by the addition of a bias term and a nonlinear activation function σ(·).

For omics input layers beyond the first, we apply a feature-wise transformation to adjust individual features prior to integration with other omics layers. This is achieved through the identity-based mapping function:


(2) $$g(X_{l})=X_{l}\cdot (I⊙D_{l}).$$

Equation (2) defines a feature-wise transformation that preserves the dimensionality of the input while enabling each feature to be independently scaled. This operation enables modulation of individual omics features prior to integration with other layers. Here, *I* is the identity matrix, and the element-wise multiplication restricts $D_{l}$ to its diagonal, enforcing a one-to-one correspondence between each input feature and its associated trainable weight.

#### 2.2.2 Layer-specific implementations

The hierarchical propagation of biological signals is modeled based on whether inter-layer interactions are additive (amplifying) or decremental (attenuating). The general formulation is:


(3) Yl={σ(g(Xl)f(Yl−1,Ml−1)+ϵ+Bl)if decremental  σ(g(Xl)·f(Yl−1,Ml−1)+Bl)if additive.

Equation (3) provides a general formulation for propagating information across layers. The additive form models amplifying biological interactions, while the decremental form captures inhibitory effects such as epigenetic suppression of gene expression. Here, ϵ is a small constant added to ensure numerical stability, prevent division by zero, and reduce sensitivity to extremely small values. The function $g(X_{l})$ transforms the input of the current layer, while $f(Y_{l-1},M_{l-1})$ incorporates the modulated output from the previous layer using biologically constrained masks.

The following layer-specific implementations reflect known biological mechanisms:

SNP layer (foundation): The initial layer transforms genetic variants ($X_{1}$) using mask-constrained connectivity, producing:


(4) $$Y_{1}=f(X_{1},M_{1}).$$

Equation (4) establishes the initial representation of genetic variation, which serves as the foundation for downstream regulatory modeling.

DNA methylation layer: Epigenetic information ($X_{2}$) is integrated with upstream genetic influences through multiplicative interactions:


(5) $$Y_{2}=\sigma \left((g(X_{2})⊙Y_{1})+B_{2}\right).$$

Equation (5) models the interaction between genetic variation and DNA methylation, reflecting how SNPs may influence epigenetic states.

Gene expression layer: Transcriptomic data ($X_{3}$) incorporates inhibitory effects of methylation through a division-based transformation:


(6) $$Y_{3}=\sigma \left(\frac{g(X_{3})}{f(Y_{2},M_{2})+\epsilon }+B_{3}\right).$$

Equation (6) captures the regulatory effect of methylation on gene expression through a division-based formulation, representing potential inhibitory relationships.

Pathway layer (GO-BP and KEGG): The pathway layer, as shown in Equation (7), aggregates gene-level representations based on predefined biological annotations without introducing new input features:


(7) $$Y_{4}=f(Y_{3},M_{3}).$$

Together, Equations (1–7) define the hierarchical propagation of biological signals across omics layers, enabling structured integration of genetic, epigenetic, and transcriptomic information within the HINN framework.

#### 2.2.3 Fully connected component and demographic integration

To provide additional flexibility in representation learning alongside sparse biological connections, we incorporate auxiliary fully connected branches at intermediate stages of the network. These dense branches are concatenated with hierarchical representations to enhance feature propagation.

The integrated pathway-level representation is then passed through a series of dense blocks consisting of batch normalization, dense layers, and dropout for regularization. Hyperparameters including layer widths, activation functions, regularization strength, and dropout rates are tuned during model optimization.

The resulting representation is concatenated with demographic and clinical features, including APOE4 status, plasma p-Tau 181 levels, age, gender, education, and race. This combined representation is further processed through additional dense layers and mapped to a final output node with a linear activation function for cognitive score prediction.

The contributions of the auxiliary dense components and biologically structured sparse connections were evaluated through ablation analyses.

### 2.3 Baseline machine learning models

To evaluate the predictive performance of HINN, we compared it against several methods, including L1, support vector machine (SVM), random forest (RF), NN, PGNN, and GOAT. Classical ML models were included to provide strong non-deep learning baselines. L1 was used to enforce sparsity and perform implicit feature selection. SVM was employed to capture nonlinear relationships through kernel functions, while RF leveraged ensemble learning to model complex feature interactions and reduce overfitting. NN was implemented to serve as a deep learning baseline without explicit biological structure. The list of hyperparameters tuned for each baseline model is shown in Table S1, available as supplementary data at *Bioinformatics* online.

The PGNN baseline was designed to assess the contribution of hierarchical integration. Unlike HINN, which models sequential cross-omics relationships, PGNN maps each omics modality independently into a shared pathway space using predefined sparse connectivity matrices (Fig. S1B, available as supplementary data at *Bioinformatics* online). The resulting pathway-level representations were combined via element-wise aggregation to form a unified pathway layer. All downstream components were kept identical to HINN, ensuring that differences in performance are attributable to the integration strategy.

To further benchmark HINN, we implemented GOAT. SNP, DNA methylation, and gene expression measurements were aligned at the gene level and represented as multi-channel node features, where each gene constituted a graph node and the three omics modalities formed the node feature vector. A gene–gene graph was constructed based on pathway co-membership. The model consisted of stacked multi-head graph attention layers followed by global pooling (Fig. S1C, available as supplementary data at *Bioinformatics* online). To ensure a fair comparison, GOAT was trained using the same gene set and evaluation pipeline as HINN.

### 2.4 Model training and evaluation

Performance of the models was evaluated using normalized mean squared error (MSE*) (Müller-Plath and Lüdecke 2024), defined as


(8) $${\text{MSE}}^{*}=\frac{\frac{1}{n}\sum_{i=1}^{n}{\left(y_{i}-x_{i}\right)}^{2}}{{\left(\bar{y}-\bar{x}\right)}^{2}+{\left(S_{x}+S_{y}\right)}^{2}}$$

where $x_{i}$ and $y_{i}$ denote predicted and observed values, $\bar{x}$ and $\bar{y}$ are their means, and $S_{x}$ and $S_{y}$ are their standard deviations. Lower values indicate better performance. Normalized mean absolute error (MAE*) and normalized root-mean square error (RMSE*) description and results are provided in the Supplementary material, available as supplementary data at *Bioinformatics* online.

Hyperparameters for all models (L1, SVM, RF, NN, PGNN, GOAT, and HINN) were optimized using five-fold cross-validation on the training data, and the best-performing configuration was selected for subsequent analyses. The genomic window used to define SNP-to-CpG connections was also selected exclusively within the training data. Candidate window sizes of 0.5, 1, and 1.5 Mb were evaluated using five-fold cross-validation with the same cross-validation splits and fixed HINN configuration across all three window sizes. For each cognitive outcome, the candidate windows were ranked according to their mean cross-validated MSE*, with lower MSE* assigned a better rank. Ranks were then averaged across the four cognitive outcomes, and the window with the lowest average rank was selected for all subsequent analyses.

For the final held-out test evaluation, the model was trained on the full training set and evaluated on the held-out test set in 10 independent runs with different random seeds. Performance is reported as the mean ± standard deviation across these 10 test-set evaluations.

### 2.5 Model interpretation and feature importance

Feature importance was computed using DeepLIFT (Shrikumar *et al.* 2017). Scores were aggregated across repeated runs, and the mean importance per feature was used for ranking. For each modality, the top 25 features were selected. Features consistently appearing in at least three of four cognitive outcomes were prioritized. Biological relevance was further assessed using Spearman correlation between selected features and cognitive scores across all samples.

## 3 Results

### 3.1 Biologically guided feature selection

We constructed a biologically informed multi-omics feature space by integrating genetic, epigenetic, and transcriptomic data through a structured pipeline. GWAS performed on the training set identified SNPs associated with cognitive outcomes, resulting in a total of 90 SNPs common across all four cognitive assessments for downstream analysis (adjusted P≤.01; Fig. S2, available as supplementary data at *Bioinformatics* online).

To capture regulatory relationships between omics layers, CpG sites located within genomic windows surrounding these SNPs and mapping to gene promoter regions were selected. We evaluated three window sizes (i.e. 0.5, 1, and 1.5 Mb upstream/downstream of SNPs) to assess the impact of genomic distance on model performance.

Feature construction was carried out by mapping CpG sites to genes and subsequently linking genes to functional annotations derived from GO-BP terms and KEGG pathways. To reduce potential bias, all GO terms and pathways associated with the genes were retained without enrichment filtering. To reduce redundancy arising from the hierarchical structure of GO, broader parent GO terms were removed when a corresponding child term was also present, thereby retaining the more specific biological annotations.

The resulting feature sets differed substantially across genomic window sizes used to map SNPs to CpG sites. For the 0.5 Mb window, the final feature set included 25 SNPs, 2220 CpG sites, 679 gene expression probes corresponding to 232 genes, and 4145 GO-BP terms and KEGG pathways. Expanding the window to 1 Mb increased the features to 30 SNPs, 4292 CpG sites, 1196 probes, and 437 genes. The 1.5-Mb window further expanded the feature space to 31 SNPs, 6139 CpG sites, 1733 probes, and 613 genes. Although the number of retained genes increased with larger window sizes, the total number of GO-BP and KEGG features remained the same (i.e. 4145), as all pathway annotations associated with the selected genes were retained and no enrichment-based filtering was applied.

Different HINN models were constructed based on the feature set for each genomic window and cognitive outcome, and performance was assessed using MSE*, with lower values indicating better predictive accuracy. As shown in Fig. 2a, the three genomic windows were evaluated using five-fold cross-validation exclusively within the training data. The 0.5-Mb window produced the lowest mean MSE* for ADAS11 (0.556), while the 1.5-Mb window produced the lowest mean MSE* for MMSE (0.438). The 1-Mb window produced the lowest mean MSE* for MoCA (0.483) and RAVLT.Immediate (0.436). To select a common window across outcomes, window sizes were ranked by mean cross-validated MSE* for each outcome, and the ranks were averaged across outcomes. The 1-Mb window achieved the lowest average rank (1.50), compared with 2.25 for both the 0.5- and 1.5-Mb windows, and was therefore selected for all subsequent analyses.

**Figure 2:**
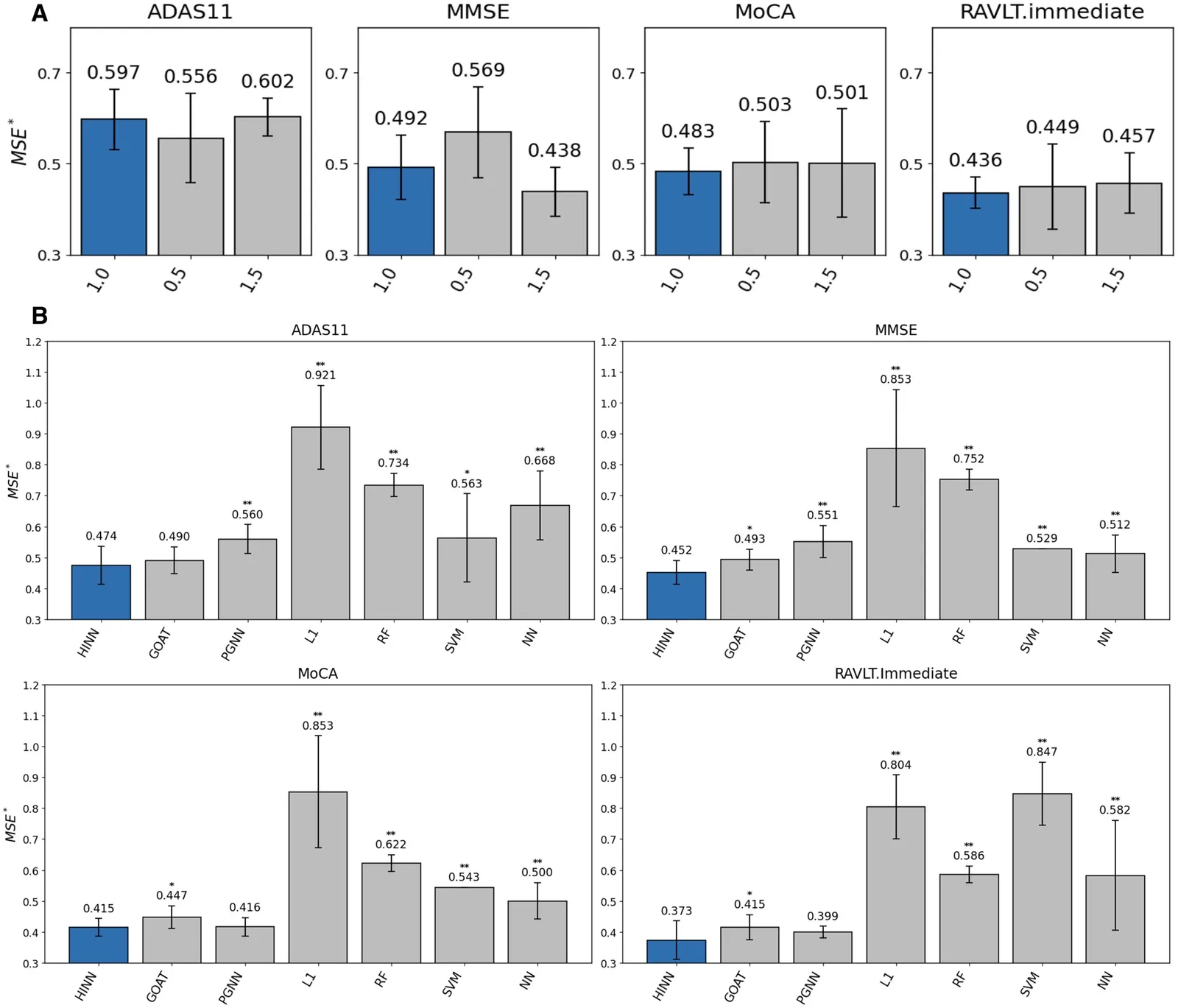
Model performance and feature set comparison using MSE*. (a) MSE* for HINN using 0.5-, 1-, and 1.5-Mb genomic windows across four cognitive outcomes, estimated using five-fold cross-validation exclusively within the training set. Error bars represent standard deviation across the five validation folds. Candidate windows were ranked by mean cross-validated MSE* for each outcome, and ranks were averaged across outcomes. The 1-Mb window achieved the lowest average rank (1.50), compared with 2.25 for both the 0.5- and 1.5-Mb windows, and was selected for subsequent analyses. (b) Performance comparison of HINN against baseline models, including L1, SVM, RF, NN, PGNN, and GOAT, using the selected 1-Mb window. Statistical significance was assessed using one-sided Welch’s *t*-tests based on summary statistics (mean ± SD across runs), testing whether HINN achieved lower MSE* than the compared models. Significance levels are indicated as (*) and (**). Error bars represent the standard deviation across repeated runs.

### 3.2 Model performance comparison

Next, we evaluated the predictive performance of HINN and baseline models using the feature set derived from the 1-Mb genomic window. Figure 2b compares HINN against baseline models, including classical ML (L1, SVM, RF), NN, a PGNN, and a graph attention-based model (GOAT). HINN achieved the lowest mean MSE* across all four cognitive outcomes. For ADAS11, HINN showed significantly lower MSE* than PGNN, L1, RF, SVM, and NN, while its difference from GOAT was not statistically significant. For MMSE, HINN significantly outperformed all baseline models. For MoCA and RAVLT.Immediate, HINN significantly outperformed GOAT and the traditional ML and NN baselines, whereas its differences from PGNN were not statistically significant.

Overall, HINN achieved the lowest mean MSE* for all outcomes and generally performed better than PGNN, GOAT, and the traditional models. Its advantage over PGNN was clearer for ADAS11 and MMSE, while their MSE* values were similar for MoCA and RAVLT.Immediate. In terms of MAE*, PGNN had the lowest mean MAE* for ADAS11 and MMSE, HINN performed best for RAVLT.Immediate, and the difference for MoCA was small (Fig. S3, available as supplementary data at *Bioinformatics* online). The RMSE* results followed the MSE* pattern because RMSE* is the square root of MSE* (Fig. S4, available as supplementary data at *Bioinformatics* online). These results suggest that PGNN may have lower average absolute errors for some outcomes, but HINN is more effective than other methods to avoid large errors.

### 3.3 Ablation studies

To evaluate the contribution of different components of the HINN architecture, we conducted a series of ablation experiments in which specific inputs, architectural modules, or connectivity patterns were removed or modified. Model performance was compared using MSE* values, as shown in Fig. 3. MAE* and RMSE* are reported in Figs S5 and S6, available as supplementary data at *Bioinformatics* online. Across these experiments, the ablation results indicate that HINN benefits from the integration of multiple omics layers and biologically structured connections. Removal of individual inputs or disruption of the sparse connectivity structure increased prediction error for most cognitive outcomes, supporting the contribution of both multimodal input integration and biologically informed information propagation.

**Figure 3:**
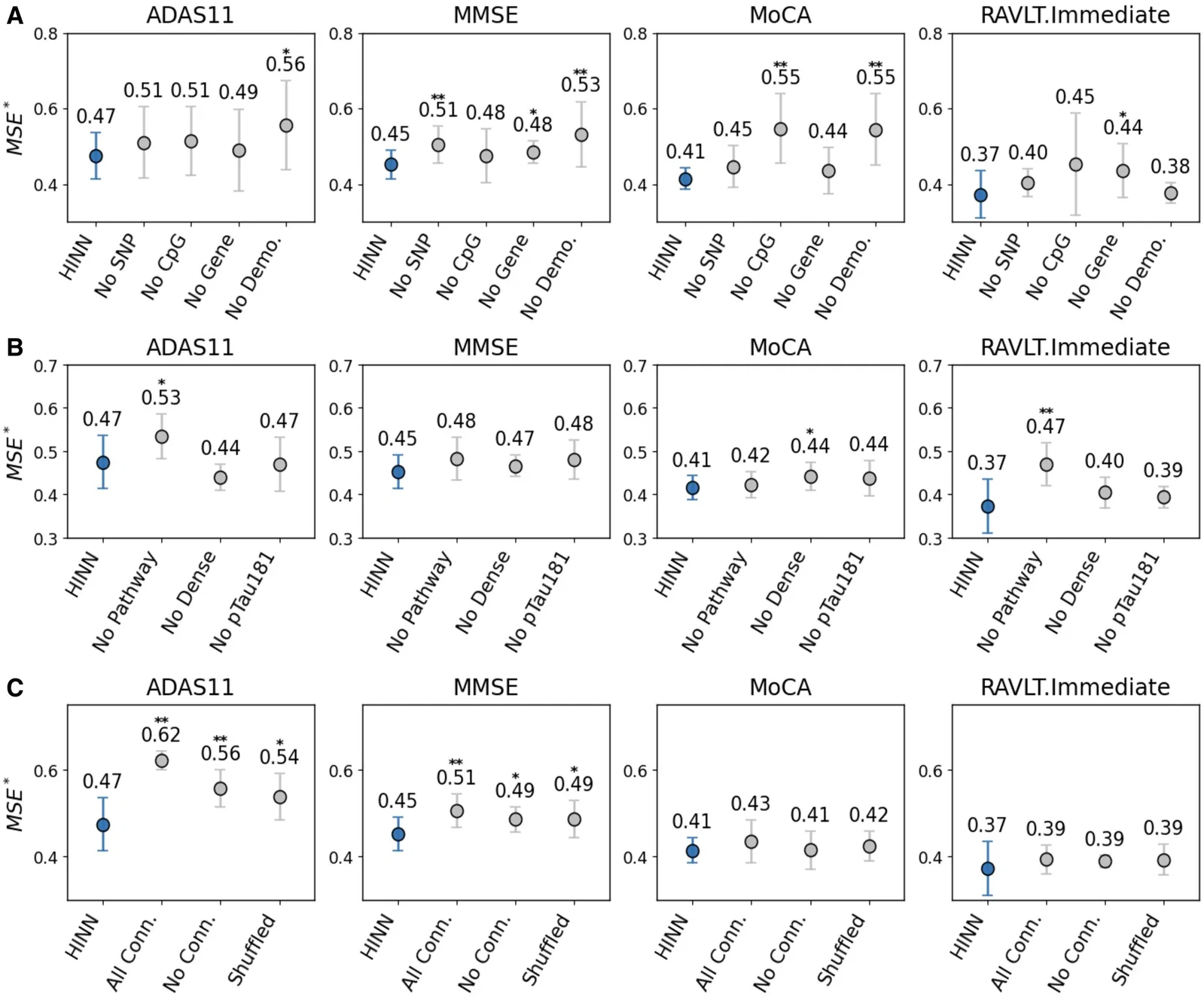
Ablation analysis of HINN components and connectivity structure with MSE* on *y*-axis. (a) Impact of removing individual input modalities, including SNP, CpG, gene expression, and demographic (Demo.) features. The full HINN model consistently achieves the lowest prediction error, while removal of any modality generally increases MSE*. (b) Impact of architectural modifications, including removal of dense connections, the pathway layer, and p-Tau 181. Performance differences are generally modest, with outcome-specific variations observed across cognitive scores. (c) Effect of connectivity structure. Replacing biologically informed connectivity with fully connected (All Conn.), no-connection (No Conn.), or randomly shuffled configurations produced outcome-dependent changes in MSE*, with the clearest degradation observed for ADAS11 and MMSE. Error bars represent standard deviation across repeated runs. Statistical significance was assessed using one-sided Welch’s *t*-tests based on summary statistics (mean ± SD across runs), testing whether the full HINN model achieved lower MSE* than ablated models. Significance levels are indicated as P<.05 (*) and P<.01 (**).

#### 3.3.1 Effect of removing an input

Figure 3a shows the impact of removing individual input modalities. The full HINN model achieved the lowest MSE* for all four cognitive outcomes. Removing SNPs, CpG sites, gene expression, or demographic variables increased prediction error, indicating that each input layer contributed complementary predictive information. The removal of demographic variables produced the largest increase in error for ADAS11, MMSE, and MoCA, highlighting the importance of demographic features in predicting these cognitive scores. For RAVLT.Immediate, the removal of demographic variables produced a smaller change relative to the other outcomes, suggesting that omics-derived features may capture a larger proportion of the predictive signal for this task.

#### 3.3.2 Effect of architectural modifications

Figure 3b shows the effect of removing selected architectural components from HINN. Removing pathway-level aggregation increased the MSE* for all four cognitive outcomes, with significant increases for ADAS11 and RAVLT.Immediate, indicating that pathway-level aggregation contributes consistently to predictive performance.

Removing auxiliary dense connections increased the MSE* for MMSE, MoCA, and RAVLT.Immediate, with a significant increase for MoCA. For ADAS11, removing dense connections produced a small, nonsignificant decrease in MSE*. Thus, dense connections improved or maintained performance for most outcomes, while their contribution to ADAS11 was limited.

Removing p-Tau181 produced smaller changes. Performance was similar to the full model for ADAS11 but worse for MMSE, MoCA, and RAVLT.Immediate. Overall, pathway-level aggregation showed the most consistent contribution, whereas dense connections and p-Tau181 had more cognitive score-specific effects.

#### 3.3.3 Effect of connectivity structure

Figure 3c shows the effect of modifying the connectivity structure across omics layers. Replacing the biologically informed sparse connections with fully connected, no-connection, or randomly shuffled connectivity increased the MSE* for most outcomes, with the largest degradation observed for ADAS11. The fully connected model performed worst for ADAS11 and also increased error for MMSE, suggesting that unconstrained connectivity introduces non-informative interactions.

For both the no-connection and randomly shuffled configurations, the increase in MSE* relative to HINN was most evident for ADAS11 and MMSE, whereas performance for MoCA and RAVLT.Immediate was closer to HINN. In the no-connection configuration, explicit biologically informed cross-omics links were removed, although information flow was still possible through the auxiliary dense nodes. The randomly shuffled configuration preserved the sparsity level but disrupted the biological organization of the connections. These results suggest that the lower error observed for HINN in ADAS11 and MMSE is associated with biologically informed sparse connectivity rather than sparsity alone or indirect information flow through dense nodes.

The MAE* results showed a different pattern: some connectivity ablations achieved slightly lower mean MAE* than HINN for ADAS11, MMSE, and MoCA, whereas HINN performed best for RAVLT.Immediate. This likely reflects the lower sensitivity of MAE* to large residuals, while MSE* and RMSE* penalize such errors more strongly. Thus, the biologically informed connectivity may primarily help reduce large prediction errors, although it does not consistently yield the lowest MAE* across all outcomes.

### 3.4 Biological interpretation of predictive features

To gain insight into the biological relationships captured by HINN, we analyzed feature importance scores derived using DeepLIFT (Shrikumar *et al.* 2017). We identified an HINN-prioritized multi-omics path involving rs116557230, cg24041822, and the *SOCS4* expression probe 11739981_s_at, which were ranked among the most influential features across cognitive measures (Fig. 4).

**Figure 4:**
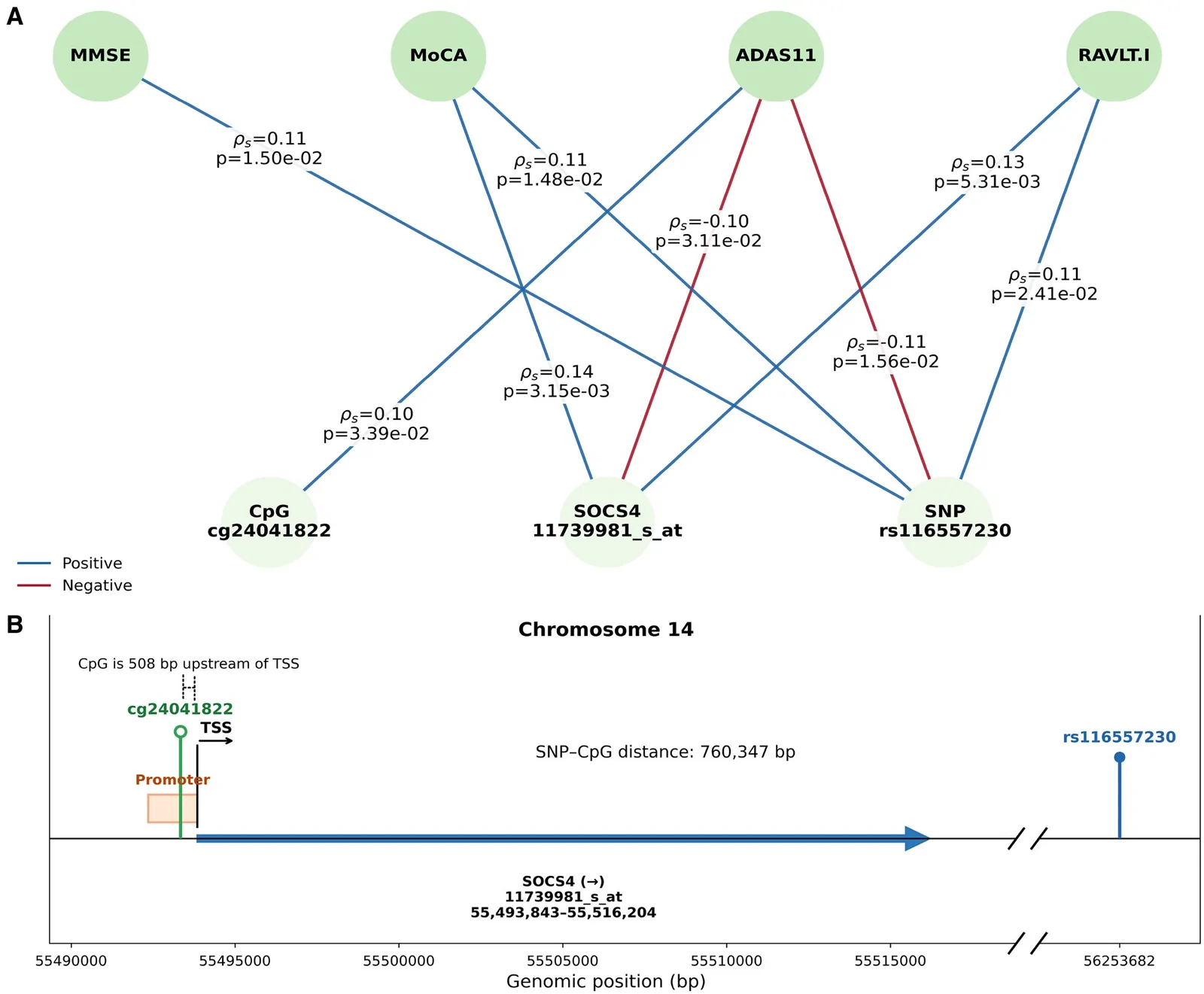
Biological interpretation of top predictive features identified by HINN. (a) Multi-omics feature cascade linking SNP (rs116557230), CpG site (cg24041822), and gene expression probe (11739981_s_at: *SOCS4*) with cognitive outcomes. Edges represent significant Spearman correlations, with the direction and strength of association indicated by the reported correlation coefficients and corresponding p-values. (b) Genomic proximity of rs116557230 and cg24041822 and the location of cg24041822 within the promoter region of *SOCS4*. Genomic positions and distances are shown in base pairs, demonstrating that the identified features fall within the 1-Mb genomic window used for model construction.

rs116557230 showed significant associations with all four cognitive scores, with a negative correlation for ADAS11 and positive correlations for MMSE, MoCA, and RAVLT.Immediate. This SNP was connected to cg24041822 in the HINN architecture because the CpG site was located 760 347 bp upstream from rs116557230, within the 1 Mb SNP-to-CpG genomic window used for edge construction. cg24041822 was ranked among the top features across three cognitive measures and showed a significant positive correlation with ADAS11, linking the model-prioritized SNP-to-CpG edge to a CpG site associated with cognitive decline. cg24041822 is located within a CpG island-associated regulatory region and maps to promoter-associated annotations of *SOCS4*, including TSS1500 and 5ʹUTR. cg24041822 is positioned 508 bp upstream of the *SOCS4* transcription start site (Fig. 4b).

The HINN-prioritized path further connected cg24041822 to the *SOCS4* expression probe 11739981_s_at. This probe was also ranked among the top features and showed significant positive correlations with MoCA and RAVLT. Immediate and a significant negative correlation with ADAS11. Because higher ADAS11 scores indicate worse cognition, whereas higher MoCA and RAVLT. Immediate scores indicate better cognition, the direction of the *SOCS4* associations was consistent across outcomes. The opposite directions observed for cg24041822 and *SOCS4* expression with ADAS11 are consistent with the model representation in which gene expression values were influenced by DNA methylation values. These results are associative and model-derived rather than evidence of methylation-mediated regulation. The relevance of *SOCS4* to cognitive decline is supported by recent CRISPR screening in iPSC-derived neurons, which identified CRL5^SOCS4^ as a regulator of tau levels and tau ubiquitination and linked this complex to resilience in tauopathies (Samelson *et al.* 2026).

The *SOCS4* probe was connected in HINN to GO and KEGG annotations through the gene-to-pathway edge construction step. These included cytokine-mediated signaling, intracellular signal transduction, protein ubiquitination, proteasomal ubiquitin-dependent protein catabolic processes, JAK-STAT signaling, insulin signaling, and type II diabetes mellitus. These annotations place the *SOCS4* signal within immune-inflammatory, proteostatic, and metabolic processes previously implicated in neurodegeneration (Jain *et al.* 2021, Ab-Hamid *et al.* 2023). In this study, these annotations provide functional context for the HINN-prioritized path but do not establish an experimentally validated regulatory mechanism.

Several additional CpG sites, including cg15767604, cg01514703, cg21350895, and cg27642489, were ranked among the top features across at least three cognitive measures and mapped to the same gene. Gene expression probes corresponding to *ZNF480* and *ADARB1* were also ranked among the top features. However, these CpG sites and probes did not show statistically significant correlations with the cognitive scores and were not emphasized in Fig. 4. Thus, the strongest interpretable signal in this analysis was the rs116557230-cg24041822-*SOCS4* path.

## 4 Discussion

In this study, we introduced HINN, a biologically structured deep learning framework for integrating blood-derived multi-omics data to predict cognitive performance. HINN differs from other models by using biologically informed links across SNPs, CpG sites, gene expression probes, and pathway-level annotations, allowing the model to represent cross-omics relationships rather than treating each feature as independent. This structure reduced the high-dimensional multi-omics input space while preserving interpretable connections across molecular layers. Compared with conventional ML and NN models without explicit inter-layer biological structure, HINN showed improved or comparable predictive performance across cognitive outcomes while retaining feature-level interpretability.

Ablation analyses further demonstrate that model performance arises from the integration of multiple complementary components rather than reliance on a single modality or architectural element. Removing individual input modalities increased prediction error, with demographic variables having a particularly strong effect for ADAS11, MMSE, and MoCA. Architectural ablations further showed that pathway-level aggregation contributed consistently to performance, while auxiliary dense connections and p-Tau181 had more outcome-specific effects. These results indicate that HINN benefits from combining multiple data sources with biologically structured information flow.

HINN also provided interpretable feature-level results. DeepLIFT attribution identified a model-prioritized path involving rs116557230, cg24041822, and the *SOCS4* expression probe 11739981_s_at. Each major node in this path was associated with one or more cognitive outcomes, with rs116557230 showing significant correlations across all four measures. The direction of the associations was consistent with the scoring of the cognitive tests: higher ADAS11 scores indicate greater impairment, whereas higher MMSE, MoCA, and RAVLT.Immediate scores indicate better cognition. Thus, opposite correlation directions for ADAS11 compared with MoCA and RAVLT.Immediate are expected when a feature tracks cognitive decline. These results support the interpretability of the HINN-prioritized path in regards to cognitive decline.

The identification of *SOCS4* is notable in light of recent CRISPR screening in iPSC-derived neurons, which identified CRL5^SOCS4^ as a regulator of tau levels and tau ubiquitination (Samelson *et al.* 2026). The GO and KEGG annotations connected to the *SOCS4* probe also point to cytokine signaling, JAK-STAT signaling, ubiquitin-dependent protein catabolism, insulin signaling, and type II diabetes mellitus, linking this model-prioritized signal to immune-inflammatory, proteostatic, and metabolic processes relevant to neurodegeneration (Jain *et al.* 2021, Ab-Hamid *et al.* 2023). Although our results do not establish a causal regulatory mechanism, they connect a blood-derived multi-omics signal associated with cognitive performance to a multi-omics context with independent relevance to cognitive decline.

Several limitations should be considered. First, the cross-omics edges in HINN were defined using biologically informed but proximity-based rules, including genomic distance between SNPs and CpG sites. Consequently, these edges should be interpreted as candidate regulatory relationships rather than experimentally validated interactions and may miss distal or context-specific effects. Second, although HINN demonstrates strong performance on the ADNI dataset, validation on independent cohorts is required to assess generalizability. Furthermore, the modest sample size limited our ability to perform reliable subgroup analyses, such as sex- or APOE-stratified modeling, because partitioning the data into smaller subgroups would reduce the number of training samples substantially relative to the high dimensionality of the multi-omics feature space. Third, DeepLIFT attributions and feature-outcome correlations should be interpreted as hypothesis-generating. They identify model-prioritized features associated with cognitive outcomes, but do not establish causal mechanisms.

Future work should evaluate HINN in independent blood-based multi-omics cohorts and test whether the rs116557230-cg24041822-*SOCS4* signal is reproducible across study populations. Incorporating experimentally supported regulatory maps, such as chromatin conformation or enhancer-promoter interaction data, may also improve the biological specificity of the cross-omics edges. Additional comparisons with alternative feature selection strategies would help separate the contribution of biologically guided feature construction from the contribution of the HINN architecture itself.

Overall, HINN provides a structured approach for integrating genetic, epigenetic, transcriptomic, pathway, and demographic information in cognitive prediction. By combining predictive modeling with biologically defined cross-omics connections, HINN improved interpretability while maintaining competitive performance across cognitive outcomes. These results highlight the potential of combining deep learning with biological knowledge to advance biomarker discovery for cognitive decline.

## Supplementary Material

btag741_Supplementary_Data

## Data Availability

The datasets analyzed in this study are publicly available from ADNI: https://adni.loni.usc.edu/. Source code, sample data, and instructions for HINN are available at GitHub: https://github.com/bozdaglab/HINN. The version of the code used for the analyses reported in this manuscript is archived on Zenodo: https://doi.org/10.5281/zenodo.19197057.
